# The Pathology and Treatment of Displacements of the Uterus

**Published:** 1889-03

**Authors:** 


**The Pathology and Treatment of Displacements of the Uterus.** By DR. B. S. SCHULTZE, Professor of Gynecology, Director of the Lying-in Institution, and of the Gynecological Clinic, in Jena. Translated from the German by James J. Macan, M. A., M. R. C. S., Eng., etc., and edited by Arthur V. Macan, M. B., M. Ch., etc. With one hundred and twenty illustrations. 8vo, pp. 378. New York: D. Appleton & Company. 1888.

The time has clearly passed when a candidate for a professorship of Gynecology will be asked, "How many ovariotomies have you made?" A more pertinent question at the present day would be, "How many deviated wombs have you cured?" It were well if all teaching were omitted with reference to ovarian tumors during the four (?) years' college course proper, excepting, perhaps, in regard to their pathology, and the other questions pertaining thereto, relegated to the post-graduate course. The time would be better taken up in the former, in teaching the subject so ably and clearly dealt with by the author before us. Abnormally deviated wombs are plentiful, and either by themselves, or through the disturbances that they generate in other organs, or, further, by the reflexes they produce, are a source unnumbered of woes to the women of the nineteenth century. It is a subject that it will not do to treat lightly, and cannot be condensed into 60 pp., as in a late so-called national system of Gynecology, that symposium of the American Gynecological Society, which, in two large octavo volumes, aggregates over two thousand pages altogether.

We wish it were possible, in the space at our disposal, to do Dr. Schultze's book credit, and to accord to it an analytical review. We must content ourselves, however, with noticing its general scope, and leave to the reader the enjoyment of perusing it without a preconceived judgment with regard to its contents from anything we shall say. Whoever would arrive at success in the management of displacements of the uterus, must first learn what the normal position of the organ is, if it be possible to do so. This seems to us to underlie the whole question of treatment, and it must be admitted that it is a most difficult problem to master. Opinions so vary as to the health line ot that peripatetic and fickle organ, and, moreover, the conditions so vary in different cases, that it is not strange that authorities have differed so widely in treating of this subject.

The careful study of Schultze's first two chapters, comprising 67 pp., will serve to throw additional light on this subject into many a darkened mind. In a chapter of 32 pp. following, he gives the symptoms and diagnosis of displacements of the uterus. What he says in regard to the employment of the uterine sound in this connection, is most cordially endorsed, and should form the key-note of the use of that too frequently used, nay even much abused, instrument. We quote: "In order to find out the position of the uterus, the examination of the uterine cavity with the sound is, in some cases, indispensable; *but rarely need anyone who is well skilled in bimanual digital palpation, make use of this method of investigation.*" We are firmly fixed in the belief that the injudicious use of the sound has done as much harm to women as any instrument which has ever been devised, and we have taken the liberty to italicise part of the quotation made from our author on this subject.

In Chapter IV., comprising eighteen pages, the Anatomy, Etiology and Indications for Treatment are considered. This is a clear, though perhaps all too brief, exposition of this branch of the subject.

In Part II., Special Pathology is taken up, and runs through the remainder of the book. Under this general head, our author considers all the various displacements and deviations of the uterus, including hernia and inversion, as sub-divisions of this general head. There may be some who will differ from the author in his anatomical teaching, but in the main it must be admitted that he is correct. It is difficult to make one who has been accustomed to think otherwise, admit to himself that the normal line of the axis of the healthy uterus is so nearly horizontal as Schultze holds it to be.

What our author says in regard to pessaries and their special application to particular cases, must be treasured up and referred to by all teachers in the future. His instructions about the shaping of his celluloid pessary are at once clear and mechanically correct. Whoever would master the question of displacement of the uterus, must have as thorough a knowledge of mechanics, as must a surgeon who would deal successfully with fractures and dislocations. Deprecate pessaries as we will, animadvert upon them as we may, and abuse them as we do, they are still capable of great usefulness when skilful hands adjust them to properly chosen cases. The great mistake, in our observation, is that this instrument is applied too soon, and without a proper knowledge of the topography of the case nine times out of ten. The pessary should never be applied at the first interview, nor until the patient has been properly prepared for its reception by a removal of all vaginal aud uterine tenderness, as well as all other contra-indicating conditions that may happen to be present in a particular case. Our author's directions are conservative, and yet bold; scientific, and yet urgent, on this subject.

At the end of each chapter a summary of its special points is given, and at the end of the book a general reference to the literature occurs, which makes the volume one of easy reference. A complete index closes this most interesting book, which we again regret to have noticed so imperfectly. The illustrations are chiefly diagrammatic drawings, but serve to illustrate the text in a clear and satisfactory manner.

It is a book which no teacher can be without, and every practiser of Gynecology will be ashamed not to have it on his shelves. The book is made up in the excellent quality and style that always characterize the publications of this house.

